# Editor’s choice 2018: Non-coding RNAs in hepatocellular cancer

**DOI:** 10.17179/excli2020-3300

**Published:** 2020-12-22

**Authors:** Ahmed Ghallab

**Affiliations:** 1 Forensic Medicine and Toxicology Department, Faculty of Veterinary Medicine, South Valley University, Qena, Egypt

## ⁯⁯

Each issue contains ten articles of a previous year selected by the editorial board. Articles selected from 2018 have a focus on non-coding RNAs, microsatellite instability, and apoptosis regulation in cancer. 

*• lncRNA involvement in hepatocellular carcinoma metastasis and prognosis*

Long non-coding RNAs (lncRNA) play a role in chromatin remodeling, methylation, and protein stability. Moreover, they may inactivate miRNA species. The authors summarized the molecular mechanisms of lncRNA in the pathophysiology of hepatocellular cancer (HCC) and give an overview over dysregulated lncRNA species (Abbastabar et al., 2018[1]). 

*• Microsatellite instability in colorectal cancer*

Molecular changes in colorectal cancer include microsatellite instability, chromosomal instability, and CpG island methylator phenotype (Nojadeh et al., 2018[9]). In this article, the authors comprehensively summarize microsatellite instability in colorectal cancer as well as their relevance in diagnosis and classification.

*• Survivin polymorphisms and susceptibility to prostate cancer: A genetic association study and an in silico analysis*

Survivin is known as an apoptosis inhibitor that influences the susceptibility to carcinogenesis (Karimian et al., 2018[4]). In this study, 157 patients with prostate cancer and 145 controls were analyzed. The authors identified significant associations of several survivin polymorphisms with prostate cancer. 

*• MicroRNA-567 inhibits cell proliferation, migration and invasion by targeting FGF5 in osteosarcoma*

MicroRNAs are known to play key roles in carcinogenesis and tumor development of osteosarcomas (Liu et al., 2018[6]). In the present article, the authors studied the consequences of exogenous expression of miR-567 in os-teosarcoma cells. They demonstrate that miR-567 reduced cell migration and invasion. Moreover, miR-567 reduces expression of fibroblast growth factor 5 (FGF5) and overexpression of FGF5 partially rescues the suppressive effects of miR-567. 

*• Unraveling the bioactivity of anticancer peptides as deduced from machine learning*

In recent years, much progress has been achieved in the development of peptide-based anticancer therapeutics. The authors summarize the state-of-the-art of the application of machine learning for analysis of the activity of anticancer peptides (Shoombuatong et al., 2018[10]).

*• Pesticide toxicity: a mechanistic approach*

This article presents a classification of pesticides, based on their pathophysiological effects (Lushchak et al., 2018[7]). Moreover, persistence in the environment, biotransformation, bioaccumulation and molecular mechanisms are systematically addressed. 

*• Natural products as reservoirs of novel therapeutic agents*

The authors provide an overview over historical natural therapeutic agents and current drug candidates (Mushtaq et al., 2018[8]). 

*• Alteration of hepatocellular antioxidant gene expression pattern and biomarkers of oxidative damage in diazinon-induced acute toxicity in Wistar rat: A time-course mechanistic study*

Diazinon is an organophosphate insecticide that was intensively used in the 1970s and 1980s for indoor pest control. In the present study, the authors describe the induction of liver damage by diazinom in Wistar rats (Hassani et al., 2018[3]). 

*• Hesperidin, a plant flavonoid accelerated the cutaneous wound healing in streptozotocin-induced diabetic rats: Role of TGF-ß/Smads and Ang-1/Tie-2 signaling pathways*

The authors demonstrate that the flavonoid hesperidin improves wound healing in a rat model of diabetic foot ulcers (Li et al., 2018[5]). 

*• Protective effects of hydrogen sulfide on chronic kidney disease by reducing oxidative stress, inflammation and apoptosis*

The authors used a partial rat nephrectomy model and analyzed blood pressure, creatinine clearance, as well as markers of oxidative stress and inflammation (Askari et al., 2018[2]). Hydrogen sulfoxide in the drinking water (30 µM) ameliorated progression of chronic kidney disease. 

## Conflict of interest

The author declares no conflict of interest.
